# Medication Adherence and Health Literacy in Patients with Heart Failure: A Cross-Sectional Survey in Iran

**DOI:** 10.3928/24748307-20220718-02

**Published:** 2022-07

**Authors:** Soheila Rezaei, Fatemeh Vaezi, Golnaz Afzal, Nasim Naderi, Gholamhossein Mehralian

## Abstract

**Background:**

Heart failure is a costly condition with high morbidity and mortality rates in low- and middle-income countries. Nonadherence to prescribed therapies can lead to severe problems such as poorer health outcomes, higher health care expenditures, increased hospitalizations, and even higher mortality rates in patients with advanced heart disease.

**Objective:**

The aim of the present study is to investigate medication adherence and the association between medication adherence and health literacy in Iranian patients with heart failure.

**Methods:**

This study was conducted in the heart failure outpatient clinic of Shahid Rajaee Cardiovascular, Medical, and Research Center in Tehran, Iran. Medical records and validated questionnaires were used to collect the necessary information on the survey variables, including sociodemographic characteristics, medication adherence, and health literacy, for a total of 250 patients with heart failure. Stepwise logistic regression analysis was performed to identify the variables that independently and significantly predicted medication nonadherence.

**Key Results:**

The results showed that most patients with heart failure had low medication adherence. Some factors, including gender, health literacy, and duration of illness, were associated with adherence. The study results showed a positive association between higher health literacy and better medication adherence.

**Conclusion:**

In view of the results, further studies on heart failure are needed to investigate other factors related to medication adherence and health literacy level to achieve better disease management and improve patients' treatment adherence. [***HLRP: Health Literacy Research and Practice*. 2022;6(3):e191–e199.**]

**Plain Language Summary:**

This study investigated the relationship between medication adherence and health literacy in Iranian patients with heart failure. The results showed that most patients had inadequate health literacy. Moreover, it showed a significant and positive relationship between health literacy and medication adherence.

Heart failure (HF) is an advanced heart disease and is a costly condition with high morbidity and mortality rates in low- and middle-income countries (Callender et al., 2014). To date, this health problem has affected approximately 26 million people worldwide, with prevalence rates (Savarese & Lund, 2017) ranging from 0.4% to 4.3% in the general population and from 2% to 20% in people older than age 75 years (Norton et al., 2011). The 1-year mortality rate of HF was reported to be 18.2% in Iran, a country in the Middle East (Ahmadi et al., 2016).

Currently, this health problem has a significant influence on the quality of life and life expectancy of patients worldwide. However, interventions to improve medical therapy should be considered a central component of HF treatment that reduces disease exacerbations, hospital readmissions, and mortality rates (Takeda et al., 2019; Ziaeian & Fonarow, 2016). With this in mind, several classes of medications have previously been recommended to treat HF (Wu et al., 2008). Medication adherence, the best predictor of treatment success and hospitalization (Amininasab et al., 2018; Pallangyo et al., 2020), has been defined by the World Health Organization as “the extent to which a person's behavior in terms of taking medications, following diets, or executing lifestyle changes coincides with medical or health advice” (Sabaté & Sabaté, 2003, p 136). Medication adherence among patients with chronic diseases is low at nearly 25% to 50% (Campbell et al., 2012). This can be a serious problem associated with poorer health outcomes, higher health care expenditures, higher hospitalization rates, and even death among patients with HF (Ehiri, 2000; Riegel & Knafl, 2013).

Compared to other chronic diseases, heart failure has a more substantial effect on individuals' performance in social, family, and marital relationships (Bagheri et al., 2014). Previous studies have also shown that medication adherence may be influenced by socioeconomic, pharmacotherapy-related, and disease-related factors, as well as factors related to patients and health care providers (Gast & Mathes, 2019; Mondesir et al., 2019). In addition, some individual characteristics and ethical concerns in patients could improve patients' adherence to HF medication, which theoretically can improve patients' health and reduce frequent hospitalizations (Amininasab et al., 2017). Of these factors, it has been noted that health literacy, as a vital indicator of health care expenditure and outcomes, can significantly contribute to medication adherence rates and HF control in patients.

Health literacy encompasses a set of socio-cognitive skills developed to improve individuals' access to health information through effective communication in conjunction with educational programs (Tavakoly et al., 2019). Accordingly, people with poor health literacy cannot understand written and verbal information given by health professionals, resulting in higher medical costs (Moshki et al., 2020). Low or limited health literacy has also been associated with poorer health status, higher rates of hospitalization, and greater use of emergency services (Oscalices et al., 2019; Tehrani Banihashemi et al., 2007). However, little is known about the relationship between medication adherence and health literacy in Iranian patients with HF. Against this background, this study set out to investigate medication adherence and the relationship between medication adherence and health literacy in Iranian patients with HF. The results may help familiarize health policymakers with the idea that improving health literacy and developing appropriate interventions could potentially lead to improved medication adherence in patients with HF.

## Materials and Methods

### Study Design and Participants

This cross-sectional study was conducted between March 2019 and December 2019 in the HF outpatient clinic of Shahid Rajaee Cardiovascular, Medical, and Research Center, in Tehran, Iran. This center was selected as the study site because it is a large, public referral hospital that provides patients with a wide range of cardiovascular diseases.

The criteria for enrolling participants in this study were (1) outpatients diagnosed with chronic HF with ejection fraction less than 35%, (2) patients taking medication for HF, (3) patients older than age 18 years, and (4) literate patients who were able to answer the questionnaire themselves. Exclusion criteria included (1) patients who had been diagnosed with HF for less than 1 month, (2) patients who were hospitalized and taking medication under close supervision, (3) patients who could not read and write, and (4) patients who could not answer the questionnaire due to the severity of the disease, old age, or other problems such as malignant disease. The eligible patients for the study were referred to the researcher by a cardiologist. Therefore, convenience sampling was used to select the participants. The researcher then asked the patients about their willingness to participate in the study. Before they participated in the study, written informed consent was obtained from the patients. They had the right to stop participating in the study at any time if they wished so.

### Assessment Tool Development

The study participants were asked to complete a three-part questionnaire. The first part of the questionnaire addressed sociodemographic characteristics such as age, gender, education level, social status, physical activity, history of heart disease, and the number of medications taken for heart disease or other conditions.

The second part of the questionnaire was the eight-item Morisky Medication Adherence Scale (MMAS-8) used to assess medication adherence (**Table A**). The Persian version of the MMAS has been validated in Iran and is widely used by researchers for chronic diseases such as hypertension and diabetes (Behnood-Rod et al., 2016; Laghousi et al., 2021; Moharamzad et al., 2015). To test the reliability of the assessment tool, Cronbach's alpha coefficient was used. A Cronbach's alpha value of 0.7 was obtained for the MMAS-8. This instrument was a structured self-report assessment of medication adherence.

**Table A:**
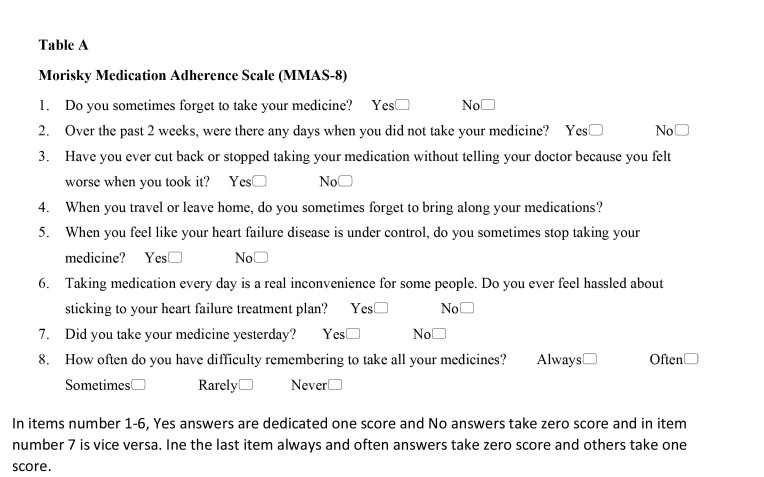
Morisky Medication Adherence Scale (MMAS-8)

1. Do you sometimes forget to take your medicine? Yes□ No□ 2. Over the past 2 weeks, were there any days when you did not take your medicine? Yes□ No□ 3. Have you ever cut back or stopped taking your medication without telling your doctor because you felt worse when you took it? Yes□ No□ 4. When you travel or leave home, do you sometimes forget to bring along your medications? 5. When you feel like your heart failure disease is under control, do you sometimes stop taking your medicine? Yes□ No□ 6. Taking medication every day is a real inconvenience for some people. Do you ever feel hassled about sticking to your heart failure treatment plan? Yes□ No□ 7. Did you take your medicine yesterday? Yes□ No□ 8. How often do you have difficulty remembering to take all your medicines? Always□ Often□ Sometimes□ Rarely□ Never□

In items number 1–6, Yes answers are dedicated one score and No answers take zero score and in item number 7 is vice versa. Ine the last item always and often answers take zero score and others take one score.

Medication adherence could be further assessed by measuring a specific medication-taking behavior with each item. This scale included seven yes/no questions and one question with a five-point Likert-type. The possible responses to the eight items included five response options ranging from *always* to *never*/*rarely*, with *always* and *often* scoring zero points and all other responses scoring one point. Finally, the MMAS-8 total score could be calculated by adding all eight individual question scores. The MMAS-8 total score could range from 0 to 8 and was categorized into three levels of adherence: high adherence (score = 8), medium adherence (score of 7 or 6), and low adherence (score <6) (Morisky et al., 2008).

The third part of the questionnaire was the Short-Test of Functional Health Literacy in Adults-16. This standard instrument consisted of 16 items to determine the level of health literacy in patients with using a Likert-type scale and was used to determine the respondents' ability to read and understand health-related information. This allowed the participants to be rated on five options (namely 4: *always*, 3: *often*, 2: *sometimes*, 1: *rarely*, and 0: *never*). The results could also be categorized as insufficient (0–32), borderline (33–48), and sufficient health literacy (49–64) (Baker et al., 1999).

### Statistical Analysis

The statistical analyses were performed using the IBM SPSS Statistics (Version 22). Visual checking was performed to prevent and catch data entry errors. Initially, descriptive statistics (namely, mean, standard deviation, frequency, and percentage) were computed to summarize the sociodemographic characteristics, medication adherence rates, health literacy levels (namely, scores). The data were also assessed for their normality using the Kolmogorov-Smirnov test.

To perform the binary stepwise logistic regression, initially, we encoded the results of the MMAS-8 questionnaire from continuous to binary variable. All of the categorical independent variables were encoded into dummy variables to allow straightforward interpretation and calculation of the odds ratio (OR) and increase the coefficients' stability and significance. To do so, we encode total scores of the Shortened-Test of Functional Health Literacy in Adults to two main categories including, inadequate and adequate health literacy, with total scores of 0 to 40 and 40 to 64, respectively.

So, factors that had a *p* value of <.05 at the univariate analysis were used in stepwise logistic regression. The analysis was performed with variable entry at a *p* value of <.05 and variable removal at a *p* value of >.1 using the OR to assess the effect of each predictor on medication adherence.

### Ethical Consideration

The Research Ethics Committee at Shahid Beheshti University of Medical Sciences in Tehran, Iran approved the study design and procedures with a code of ethics: IR.SBMU. PHARMACY.REC.1398.008. The study also complied with the Declaration of Helsinki.

## Results

### Sociodemographic Characteristics

Over the course of 9 months, 286 patients with were invited to participate in the study, and 250 of them eventually consented. The sociodemographic characteristics and medical information of the study population are shown in **Table 1**. Of the 250 participants with HF, 60% were men, and nearly 76% were between age 41 and 80 years. Nearly 73% of the participants had a lower level of education with a high school diploma or even lower. Most participants in this study (more than 90%) did not smoke or drink alcohol. About one-half of the participants had been diagnosed with HF for less than 5 years. Almost one-half of the patients were taking 6 to 10 medications.

**Table 1:**
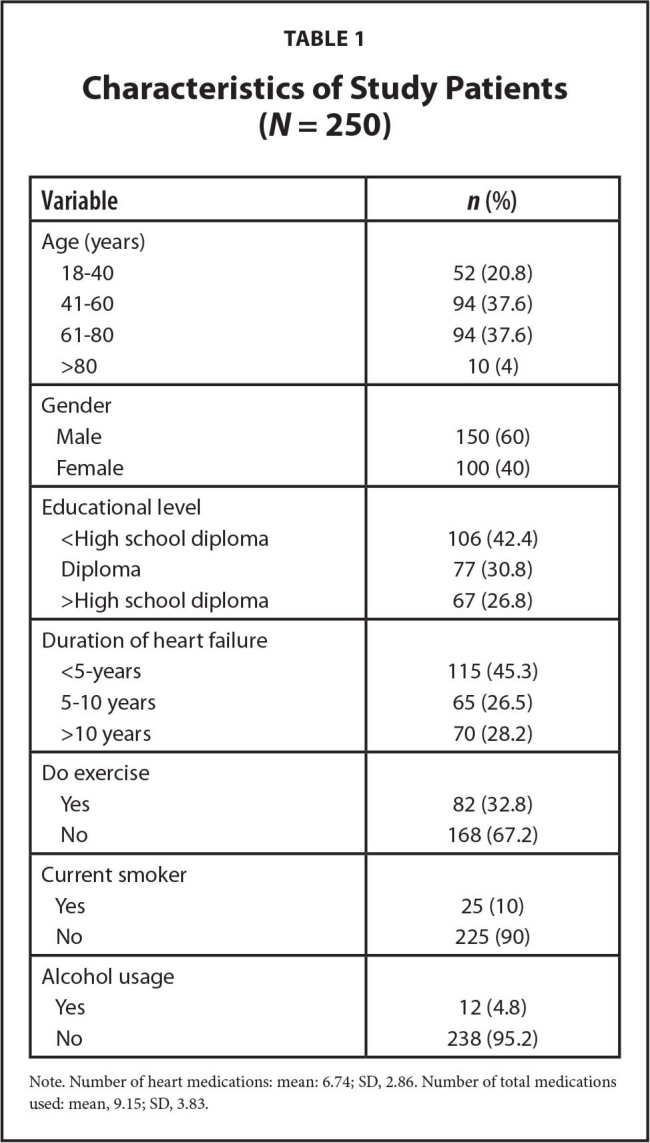
Characteristics of Study Patients (*N* = 250)

**Variable**	***n* (%)**

Age (years)	
18–40	52 (20.8)
41–60	94 (37.6)
61–80	94 (37.6)
>80	10 (4)

Gender	
Male	150 (60)
Female	100 (40)

Educational level	
<High school diploma	106 (42.4)
Diploma	77 (30.8)
>High school diploma	67 (26.8)

Duration of heart failure	
<5–years	115 (45.3)
5–10 years	65 (26.5)
>10 years	70 (28.2)

Do exercise	
Yes	82 (32.8)
No	168 (67.2)

Current smoker	
Yes	25 (10)
No	225 (90)

Alcohol usage	
Yes	12 (4.8)
No	238 (95.2)

Note. Number of heart medications: mean: 6.74; SD, 2.86. Number of total medications used: mean, 9.15; SD, 3.83.

### Medication Adherence Rates and Health Literacy Levels

**Figure 1.**
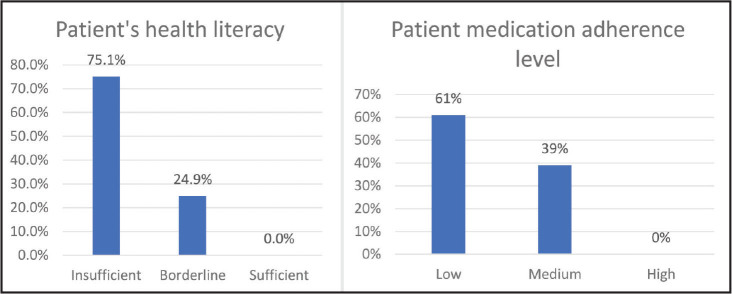
Distribution frequency of medication adherence and health literacy among patients.

Among the most common factors associated with medication nonadherence among HF patients were reasons other than forgetfulness. In other words, at baseline, 95.2% and 85.5% of the patients' answer the questions “When you feel that your illness is under control, do you ever stop taking your medication?” and “Have you ever reduced or stopped taking your medication because you felt worse after taking it?” was yes. However, only 51.8% of the patients mentioned forgetfulness as the main reason for their nonadherence behavior. According to the sum of the scores of the MMAS-8, 61% of the patients with HF had low medication adherence, and 39% of them showed moderate scores in this regard. Moreover, none of the patients had high medication adherence rates (**Figure 1**).

As shown in **Figure 1**, 75% of the participants had inadequate health literacy, and 25% were classified as having borderline health literacy. However, none of the patients had reported adequate health literacy.

### Univariate Analysis of Predictors of Medication Adherence

The following variables were identified as significant predictors of medication adherence (**Table 1**): gender (p <.05), health literacy (p <.05), less than 5 years of medical history of HF (p <.01), and the number of cardiovascular (CV) medications (*p* = .05).

### Multivariate Analysis of Predictors of Medication Adherence

According to the final logistic model shown in **Table 2** and **Table 3**, the likelihood of patient adherence to medication was significantly associated with the variables of the medical history of HF (p <.01), gender (p <.05), and health literacy (p <.05). The OR values indicate that the patients studied were likely to be 4.5 times more adherent if they had been on therapy for less than 5 years (OR 4.56; confidence interval [CI] [2.05, 10.16]). Women were approximately 1.8 times less likely to adhere than men (OR 0.55; CI [0.31, 0.98]). Finally, adherence was found to be likely to be about 3.4 times lower in patients with low health literacy than in patients with borderline health literacy (OR 0.3; CI [0.13, 0.66]).

**Table 2:**
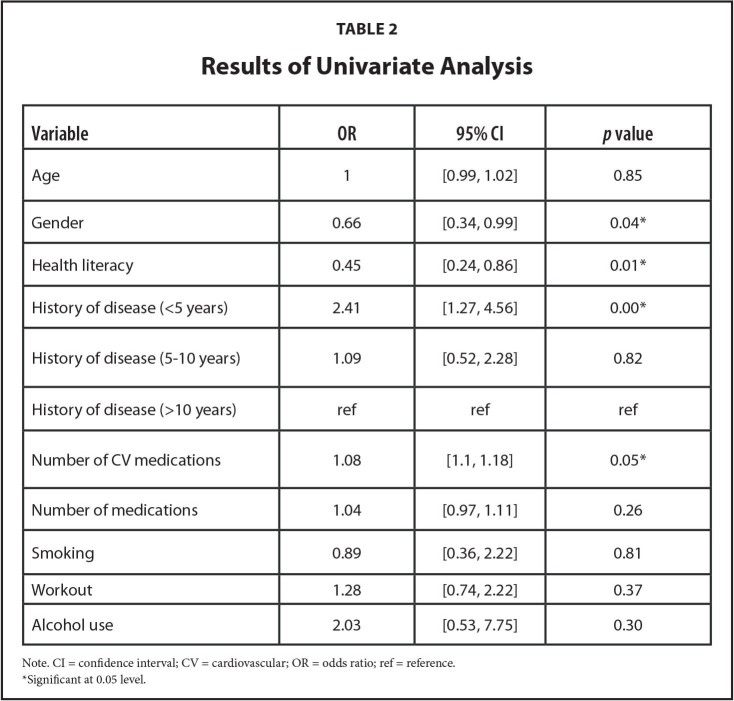
Results of Univariate Analysis

**Variable**	**OR**	**95% CI**	***p* value**
Age	1	[0.99, 1.02]	0.85
Gender	0.66	[0.34, 0.99]	0.04^*^
Health literacy	0.45	[0.24, 0.86]	0.01^*^
History of disease (<5 years)	2.41	[1.27, 4.56]	0.00^*^
History of disease (5–10 years)	1.09	[0.52, 2.28]	0.82
History of disease (>10 years)	ref	ref	ref
Number of CV medications	1.08	[1.1, 1.18]	0.05^*^
Number of medications	1.04	[0.97, 1.11]	0.26
Smoking	0.89	[0.36, 2.22]	0.81
Workout	1.28	[0.74, 2.22]	0.37
Alcohol use	2.03	[0.53, 7.75]	0.30

Note. CI = confidence interval; CV = cardiovascular; OR = odds ratio; ref = reference.

* Significant at 0.05 level.

**Table 3:**
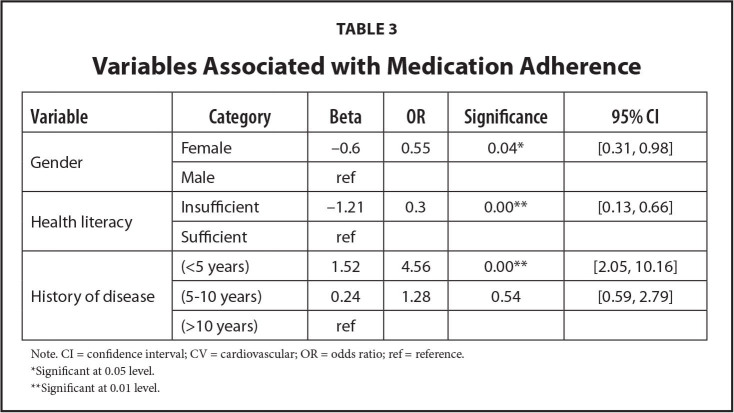
Variables Associated with Medication Adherence

**Variable**	**Category**	**Beta**	**OR**	**Significance**	**95% CI**
Gender	Female	−0.6	0.55	0.04^*^	[0.31, 0.98]
	Male	ref			
Health literacy	Insufficient	−1.21	0.3	0.00**	[0.13, 0.66]
	Sufficient	ref			
History of disease	(<5 years)	1.52	4.56	0.00^**^	[2.05, 10.16]
	(5–10 years)	0.24	1.28	0.54	[0.59, 2.79]
	(>10 years)	ref			

Note. CI = confidence interval; CV = cardiovascular; OR = odds ratio; ref = reference.

* Significant at 0.05 level.

** Significant at 0.01 level.

The Chi-square value of 43.56 with degrees of freedom equal 4, and p <.001 indicates that the final model is reliable. The probability that the independent variables are taken together does not affect the outcome variable is <0.001. The Nagelkerke R2 value was 0.28, which means that the predictors of the model explained 27.8% of the variance in medication adherence.

## Discussion

This study aimed to investigate medication adherence and the relationship between medication adherence and health literacy in Iranian patients with HF. The present results show that more than one-half of the included patients with HF had low medication adherence. Nonadherence to medication in patients with HF is a common health problem and a major concern for health professionals (Schlenk et al., 2004). Such nonadherence may therefore minimize the effectiveness of prescribed therapies and expose patients to clinical deterioration, profoundly affecting their health status (Shilbayeh et al., 2018; van der Wal & Jaarsma, 2008). Thus, assessing and exploring potential factors associated with medication adherence is a critical step toward improving clinical and health-related outcomes as a central pillar of effective pharmacotherapy in patients with HF.

The results of the univariate predictors of medication adherence show that gender, health literacy, having had the disease for less than 5 years, and the number of CV medications are among the most important predictors. Multivariate predictors also show that history of HF, gender, and health literacy are among the most important predictors of medication adherence in the population of the current study. As described in the literature, many factors can lead to nonadherence in patients with HF, including patient health literacy, which has increasingly drawn the attention of researchers to show how it influences patient adherence behaviors (Chen et al., 2014; Qobadi et al., 2015). Similarly, the available literature shows that health literacy in the general Iranian population is insufficient (Haghdoost et al., 2019). Although many studies to date have assessed patients' health literacy and examined factors associated with medication adherence (Chahardah-Cherik et al., 2018; Sanati et al., 2020; van der Laan et al., 2017), few studies have examined the relationship between medication adherence and health literacy levels in patients with HF (Noureldin et al., 2012; Oscalices et al., 2019).

Despite the presence of a theoretical association, generally, prior research on the relationship between health literacy levels and medication adherence is inconclusive yet (Gellad et al., 2011; Persell et al., 2020). Some studies have simply suggested that health literacy not only has a direct effect on adherence but may also mediate the effects of socio-cognitive skills on adherence (Geboers et al., 2015; Wannasirikul et al., 2016). Considering that the nature of the potential association between medication adherence and health literacy is not explicit, further studies need to be conducted to increase the shared knowledge and understanding of some unknown factors in this area. Therefore, this prospective cross-sectional survey was designed to estimate the medication adherence of patients with HF and its relationship with some sociodemographic variables and investigate the correlation between medication adherence and the level of health literacy in patients with HF.

The study results showed that most patients were not adherent to their medications. More than one-half of the patients also cited reasons other than forgetfulness as the main reason for nonadherence. In contrast, previous studies had reported that patients with HF did not take their medication due to forgetfulness (Aggarwal et al., 2015; Al-Ramahi, 2015). Some studies have offered various strategies to improve patient adherence to address the mentioned issue, such as adherence checks, mobile apps, automated checks, medication schedules, and text message reminders (Aggarwal et al., 2015; Al-Ramahi, 2015; Mortara et al., 2020).

“Feeling worse when taking HF medication” as well as “Taking no medication when feeling like being under control” were accordingly the main reasons for low medication adherence in this study. Apparently, the above two reasons showed that patients' medication adherence behaviors depended on their perceived emotional situation. In both cases, if they felt worse while taking HF medication and felt being under control, they were not emotionally satisfied to adhere to their medication routine. This behavior could be related to the chronic nature of the disease, the side effects of the medications, and low health literacy. To address this problem, prescribing physicians and pharmacists are recommended to communicate better with patients (Al-Ramahi, 2015; Amorha et al., 2021).

The present study results showed a significant relationship between medication adherence and patients' gender. Women were found to have lower medication adherence, while previous studies have shown otherwise (Caspard et al., 2005; Fodor et al., 2005; Lertmaharit et al., 2005). Moreover, other studies failed to find any association between gender and medication adherence (Bani Asad et al., 2014; Jin et al., 2008; Tiv et al., 2012). These differences could be related to cultures, societies, and gender roles. However, due to the conflicting results, gender may not be a good predictor of medication adherence.

Our results showed a significant relationship between the duration of the HF disease and the level of adherence, suggesting that a longer duration of HF disease has a negative impact on medication adherence. As reported in previous adherence studies, the longer the disease duration, the lower the patient adherence (Gadallah et al., 2015; Morgan et al., 2015). It could be because there is a decrease in the severity of beliefs in the need for medication and support from professionals or family members over time. Eventually, disruption of treatment control follows and causes medication adherence to decrease dramatically after several years of chronic illness.

This study found no significant association between patients' educational level and medication adherence. Although several studies have shown conflicting results regarding the relationship between medication adherence and education level (Fuster, 2012; Jin et al., 2016; Theofilou, 2011), there is a need to conduct further research on this topic to draw more reliable and consistent conclusions. Interestingly, the results of this study are consistent with previous surveys that showed no significant relationship between the number of medications are taken and adherence rates (Granger et al., 2009; Riegel & Carlson, 2002). As medications are an essential part of chronic disease management (Vermeire et al., 2001), the concept of a combination pill, or polypill, could be a successful method to improve adherence rates (Muscente & De Caterina, 2016).

This study also showed that most participants (74%) had inadequate health literacy. This is also in line with the findings of previous studies that reported low levels of health literacy among Iranians (Tavousi et al., 2016; Tehrani Banihashemi et al., 2007). Moreover, the study results clearly showed a significant relationship between medication adherence and health literacy. Previous studies in this area had found a positive relationship between higher levels of health literacy and higher medication adherence (de Souza & de Carvalho Queluci, 2014; Macabasco-O'Connell et al., 2011). For example, Nutbeam et al. (2015) reported that inadequate health literacy is a barrier for better disease management, directly related to patients' awareness of having control over their disease. Some studies have shown that health literacy is an essential factor influencing medication adherence, rehospitalization, and mortality rates in patients with HF. Most of the patients included in these studies were women (Murray et al., 2009; Oscalices et al., 2019). However, in our study, men predominated.

Although the results of several studies suggest that health literacy has a significant effect on adherence in patients, most patients in these studies had borderline or adequate health literacy (Jo et al., 2020; Noureldin et al., 2012). In our research, most of the patients had inadequate health literacy.

Therefore, pharmacists can play a crucial role in this context, as they can assess patients' health literacy and develop appropriate interventions, such as educational modules that promote disease understanding, higher medication adherence, and self-care behaviors related to HF (Chan et al., 2020).

Further studies of HF patients are needed to examine other factors related to adherence and level of health literacy to achieve better disease management and improve patient adherence.

## Study Limitations

This study had some limitations that can be considered in future surveys. First, a multicenter study is strongly recommended, although this study was conducted in a large and highly specialized center for HF patients. Second, this study did not consider the follow-up period to evaluate the association between health literacy and mortality data. Therefore, to generalize the present study results, similar surveys with larger sample sizes are emphatically suggested to improve the shared knowledge and understanding of some unknown factors in this area. Another limitation concerns self-report bias in terms of recall and social desirability. To avoid this type of bias, adherence is recommended to be measured using direct methods.

## Conclusion

The study findings highlighted that some variables, including age, level of education, social habits, and physical activity, were not associated with medication adherence in patients with HF. In multivariate analysis, the factors including gender, health literacy, and the duration of the disease were associated with medication adherence. The results also showed that more than half of the participants with HF had low medication adherence; hence, it is necessary to consider factors influencing medication adherence rates to improve patients' health status. Additionally, the findings revealed that most of the patients with HF had insufficient health literacy levels, which was associated with lower medication adherence rates.
